# Establishment and characterization of new human cell lines for recombinant therapeutic protein production

**DOI:** 10.1186/1753-6561-9-S9-P3

**Published:** 2015-12-14

**Authors:** Yuichi Inoue, Akira Iwamoto, Aiko Inoue, Hiroharu Kawahara

**Affiliations:** 1 National Institute of Technology, Kitakyushu College, Kitakyushu, 802-0985, Japan; 2 Department of Bioscience and Biotechnology, Kyushu University, Fukuoka 812-8581, Japan; 3 Department of Clinical Laboratory, Kyurin Corporation, Kitakyushu 806-0046, Japan

## Background

Recombinant therapeutic proteins are increasingly requested with advances in tissue engineering using stem cells. Human cell line is an attractive host for the production of such glycoprotein, but there are few reports on human cells for a commercial production [1]. In this study, we established new human lymphoid cell lines from peripheral blood mononuclear cells (PBMCs) by treatment with phorbol 12-myristate 13-acetate (PMA) under a non-GMP condition, and characterized them by gene and protein expression analyses.

## Experimental Approach

The human PBMCs (2 × 106 cells/ml) were cultured in 24 well plates in 12.5%FBS-ERDF medium supplemented with 10 ng/ml of IL-4 and/or IL-6 and 1 µg/ml of PMA for three months. The medium was changed every two or three days.

The gene expression of telomerase reverse transcriptase (TERT), a marker of immortalization, was examined by RT-PCR. The immunoglobulin (Ig) isotype was confirmed by ELISA. The CD markers on cell surface were detected by flow cytometry.

## Results and Discussion

Although normal human cells are difficult to be transformed by chemical reagents such as PMA, we succeeded in obtaining three human cell clones under above condition (Table 1). The key point in our transformation may be continuous stationary culture without passage. All obtained clones were able to be subcultured over one year and were found to express TERT. In addition, they produced any isotype of immunoglobulin in the medium, indicating B lymphocytes. The results of flow cytometry also supported it. Therefore, these clones may be suitable for the production of secreted human glycoprotein because mature B lymphocytes have the extensive endoplasmic reticulum. In the next step, we will establish GMP compliant new human lymphoid cell lines.

**Table 1:** Summary of cell establishment and characterization.

	Clone1	Clone2	Clone3
Culture medium for transformation	12.5%FBS-ERDF +IL-4+IL-6+PMA	12.5%FBS-ERDF +IL-6+PMA	12.5%FBS-ERDF +IL-4+IL-6+PMA
Culture medium for maintenance	12.5%FBS-ERDF (+PMA)	12.5%FBS-ERDF (+PMA)	12.5%FBS-ERDF (+PMA)
Possible period of subculture	Over 1 year	Over 1 year	Over 2 years
Mean doubling time	30 hours	30 hours	30 hours
TERT expression	Positive	Positive	Positive
Produced Immunoglobulin	IgG	IgM	IgG
B cell marker (CD19)	Positive	Positive	Positive

## Acknowledgements

This work was supported by JSPS KAKENHI Grant Number 26460167.
